# Valproic Acid: A Promising Therapeutic Agent in Glioma Treatment

**DOI:** 10.3389/fonc.2021.687362

**Published:** 2021-09-10

**Authors:** Wei Han, Wei Guan

**Affiliations:** ^1^Department of Neurosurgery, The Third Affiliated Hospital of Soochow University, Changzhou, China; ^2^Department of Neurosurgery, Huashan Hospital, Fudan University, Shanghai, China

**Keywords:** valproic acid (VPA), glioma, anti-tumor effects, synergistic effects, clinical trials

## Abstract

Glioma, characterized by infiltrative growth and treatment resistance, is regarded as the most prevalent intracranial malignant tumor. Due to its poor prognosis, accumulating investigation has been performed for improvement of overall survival (OS) and progression-free survival (PFS) in glioma patients. Valproic acid (VPA), one of the most common histone deacetylase inhibitors (HDACIs), has been detected to directly or synergistically exert inhibitory effects on glioma *in vitro* and *in vivo*. In this review, we generalize the latest advances of VPA in treating glioma and its underlying mechanisms and clinical implications, providing a clearer profile for clinical application of VPA as a therapeutic agent for glioma.

## 1 Introduction

Glioma, originating from the neuroglial stem or progenitor cells, is the most prevalent and aggressive primary intracranial tumor (1, 2). Clinically, the standard therapy for glioma patients includes surgical intervention and adjuvant radiotherapy and chemotherapy (3). However, owing to its infiltrative growth and resistance to comprehensive treatment, the mortality and recurrence rate of glioma patients are still high, leading to poor prognosis (4). Therefore, it is crucial to summarize latest advances in glioma treatment and grope for promising investigational directions.

**Figure 1:**
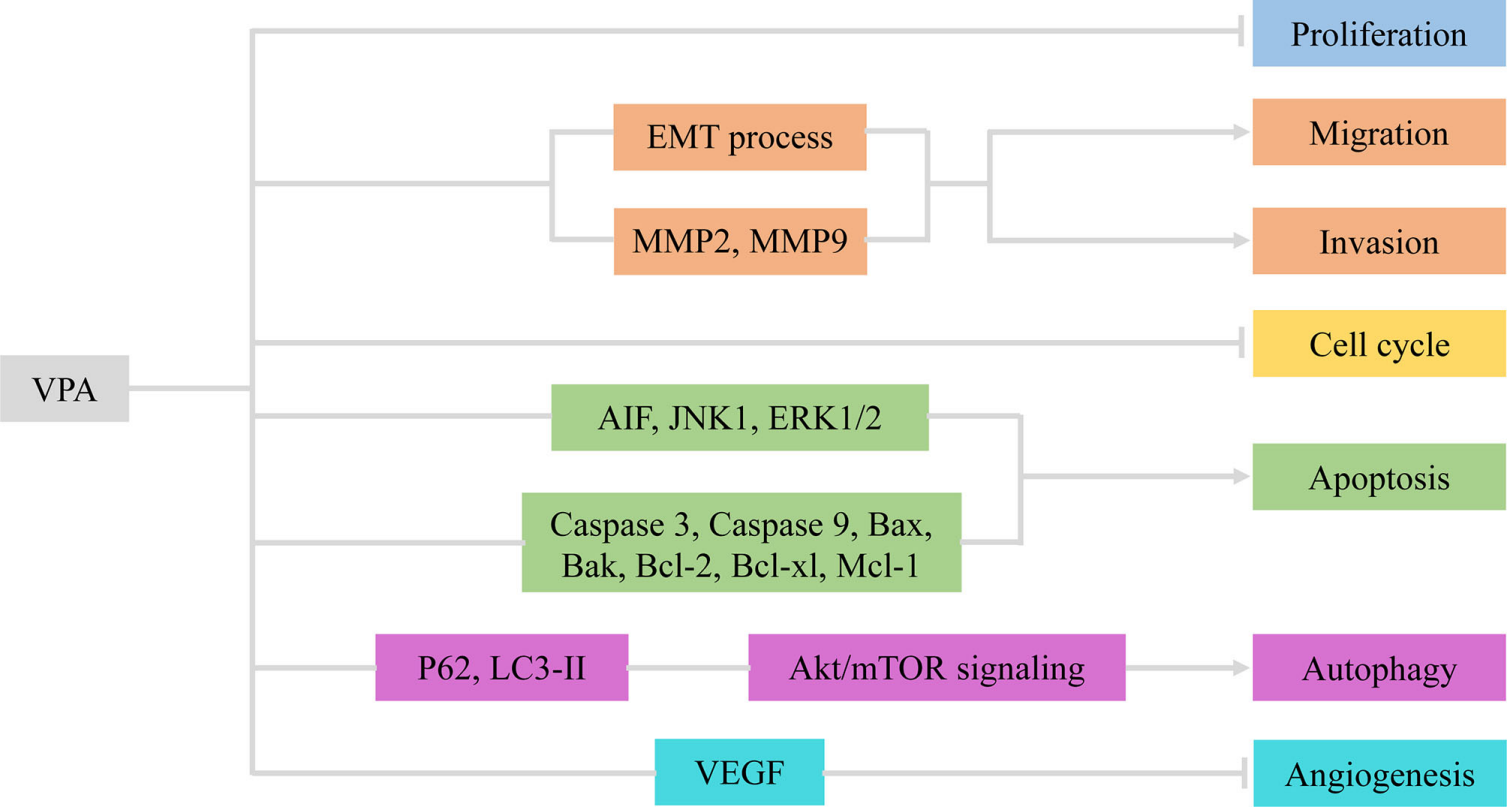
VPA exerted therapeutic effects *via* involvement with cellular activities of glioma. VPA, valproic acid; EMT, epithelial–mesenchymal transition; MMP2/9, matrix metalloproteinase 2/9; AIF, apoptosis-inducing factor; VEGF, vascular endothelial growth factor.

**Figure 2:**
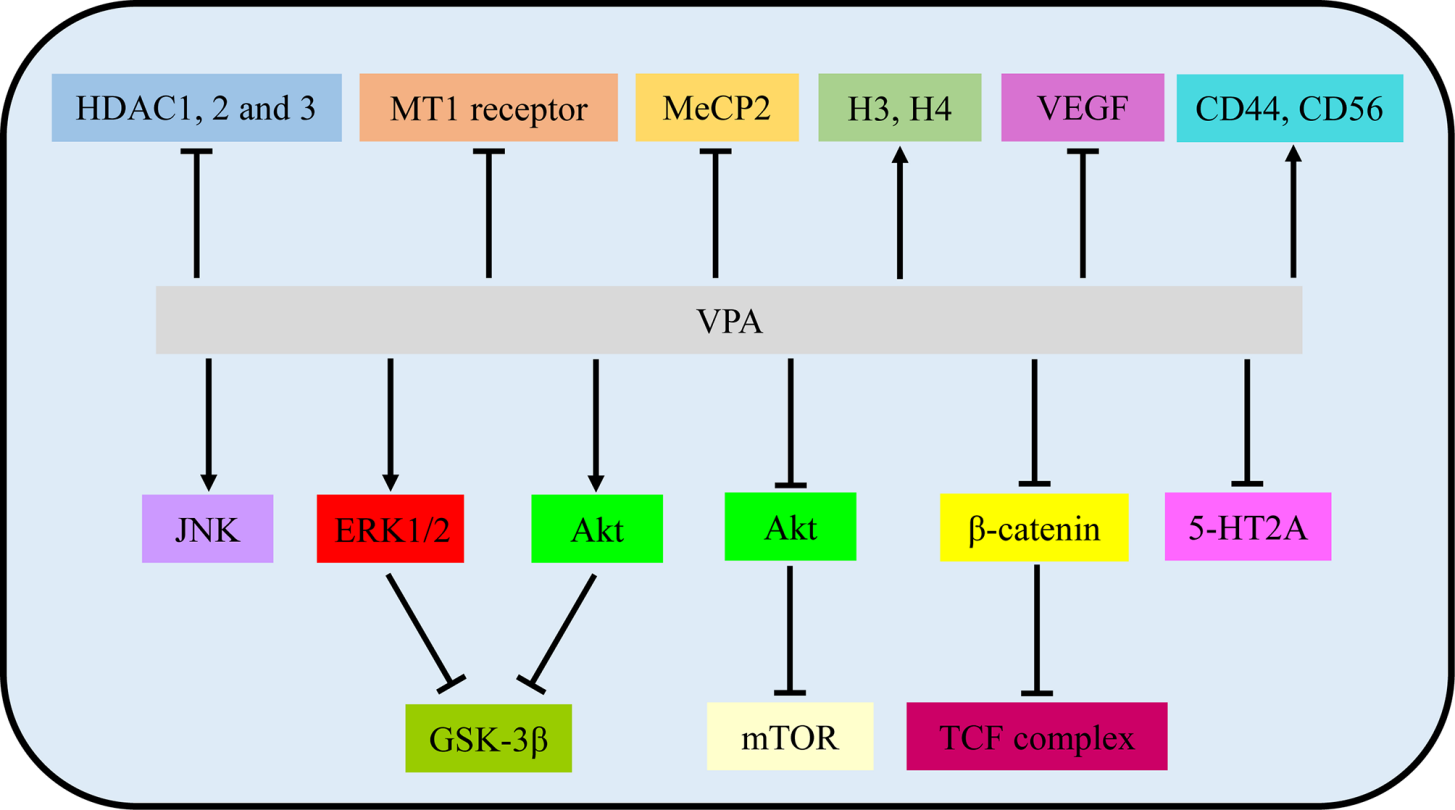
VPA exerted therapeutic effects *via* involvement with signaling pathways and molecular targets. VPA, valproic acid; HDAC, histone deacetylase; MT1, MT1: melatonin 1; MeCP2, methyl CpG binding protein 2; H3/4, histone 4; VEGF, vascular endothelial growth factor.

**Figure 3:**
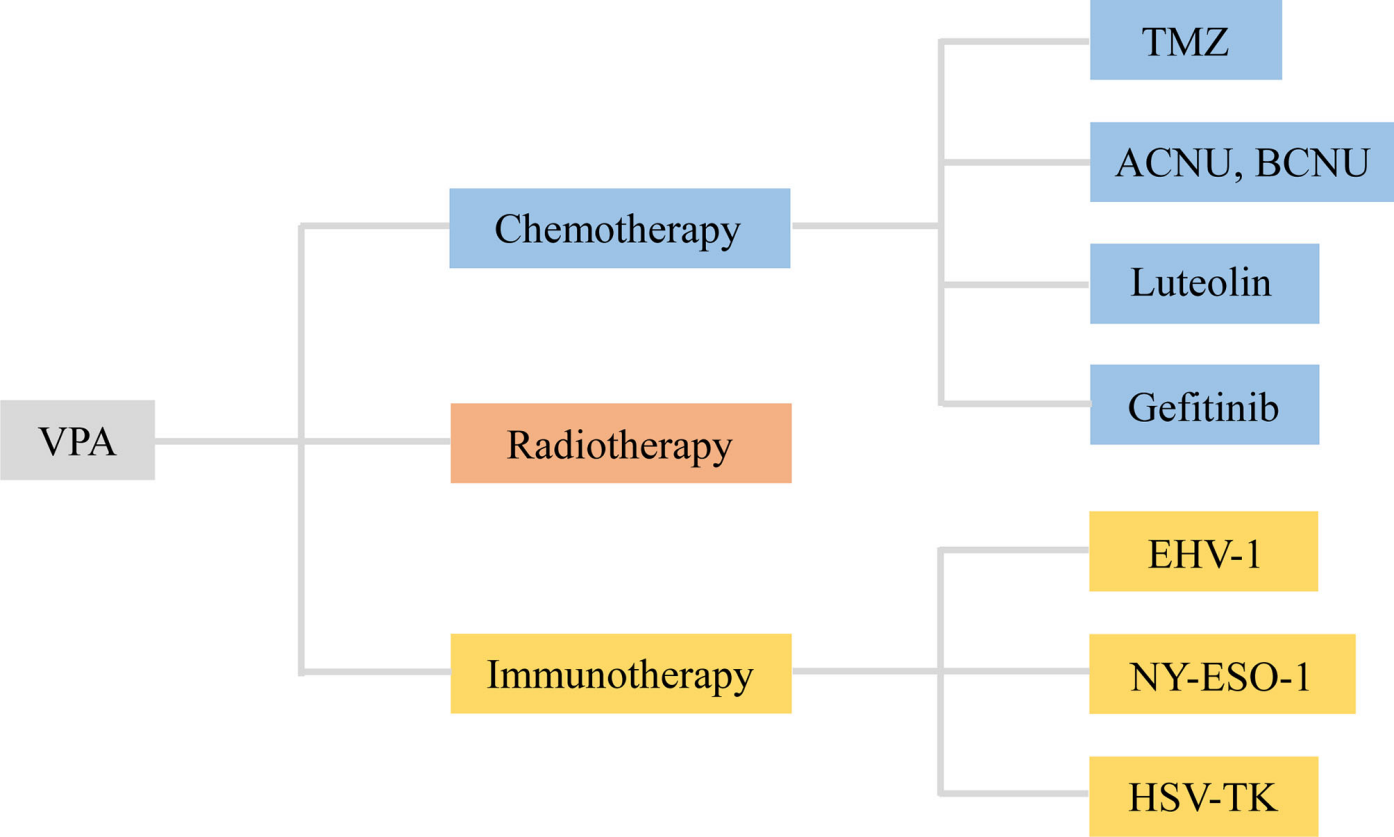
VPA served as an adjuvant agent in chemotherapy, radiotherapy, and immunotherapy of glioma. VPA, valproic acid; ACNU, nimustine; BCNU, 1, 3-bis (2-chloroethyl)-1-nitrosourea; EHV-1, equine herpesvirus type 1; HSV-TK, herpes simplex virus type I thymidine kinase.

Valproic acid (VPA), one of the most common histone deacetylase inhibitors (HDACIs), is known as an anticonvulsant and mood-stabilizing drug clinically (5). Gathering evidence have manifested that VPA directly or synergistically exerted anti-tumor effects on various solid tumors (6, 7). For instance, VPA suppressed gastric cancer cell proliferation and induced autophagy through HDAC1/PTEN/Akt signaling (8). Similarly in breast cancer, the inhibitory role of VPA is mainly reflected in cellular proliferation, cell cycle, and apoptosis *via* Hsp70 acetylation (9), while in most cancers, VPA tended to serve as an adjuvant drug for chemotherapy, radiotherapy, and other therapies. In lung cancer, VPA and arsenic trioxide markedly potentiated cell death *in vitro* and *in vivo* (10). VPA also modulated invasion capability targeting MMP-1, MMP-3, and MMP-13 to enhance the radiotherapy effect of breast cancer cells (11). Furthermore, VPA sensitized pancreatic cancer cells to NK cell-mediated lysis by upregulating MICA and MICB *via* PI3K/Akt signaling pathway (12). Currently, various studies have ascertained that application of VPA is effectively involved in glioma treatment (13–15). In this review, we generalize the latest advances of VPA in treating glioma and its underlying mechanisms (**Figures 1**–**3** and **Tables 1**, **2**) and clinical implications (**Table 3**), providing a clearer profile for clinical application of VPA as a therapeutic agent for glioma.

**Table 1:** VPA was involved in cellular activities of glioma through signaling pathways and molecular targets.

Glioma cell lines	Concentration and Duration (VPA)	Targets	Biological activities	Ref.
U251, SNB19	2 mM/48 h	Akt/mTOR signaling	↓cell viability, ↑apoptosis, ↑autophagy	(13)
GBM2, G144	2 mM/96 h	Wnt/β-catenin signaling	↓proliferation, ↓invasion	(16)
U87	4 mM/72 h	ERK/Akt signaling	↓cell viability, ↑apoptosis	(17)
A172, T98G	A172:0–50 mM/24–48 h T98G:0–100 mM/24–48 h	MAPK signaling	↑cytotoxicity, ↓invasion, ↑apoptosis	(18)
U87, U251, U343	0–5 mM/24–96 h	VEGF	↓angiogenesis	(19)
U87MG, SF295, T98G	0–10 mM/96 h	ERK signaling	↓cell viability, ↑cell-cycle arrest, ↑autophagy	(20)
C6, U373	0–10 mM/24–72 h	H4	↓proliferation, ↓migration, ↑cell-cycle arrest	(21)
C6	0.5 mM/1 or 7 days	MT1 receptor, MeCP2, HDAC1, 2, 3	\	(22)
A172, U373, U138, U87, SW1783	2 mM/96 h	H3 and H4	↓proliferation	(23)
C6	5 mM/24 h or 48 h	MT1 receptor, BDNF, GDNF, HDACs	↑neuroprotection	(24)
C6	100 mg/ml/20 h	5-HT2A receptor signaling	\	(25)
A172, 85HG66	0–1 mM	CD56, CD44	↓proliferation	(26)

VPA, valproic acid; VEGF, vascular endothelial growth factor; H3, histone 4; MT1, melatonin 1; MeCP2, methyl CpG binding protein 2; HDAC, histone deacetylase; BDNF, brain-derived neurotrophic factor; GDNF, glial cell-derived neurotrophic factor.

↓ -Inhibition, ↑ -Promotion.

**Table 2:** VPA served as an adjuvant agent in glioma treatment *via* involvement with cellular activities and biological targets.

Glioma cell lines	Concentration and Duration (VPA)	Other Therapeutic Agents	Targets	Biological Activities	Ref.
U251, LN229, SNB19	1 mM/48 h	Luteolin/20 μM/48 h	Akt signaling	↓cell viability, ↓migration, ↓cell-cycle progression, ↑apoptosis	(27)
G1, G2, G3	1 mM/72 h	TMZ/0–500 mM/72 h ACNU/0–250 μg/ml/72 h	MGMT	↓cell viability, ↑apoptosis	(28)
U87, T98G	1 mM/24 h	Gefitinib/10 μM/24 h	LKB1/AMPK signaling	↓cell viability, ↑apoptosis, ↑autophagy	(29)
U251, SNB19	1 mM/24 h	EHV-1/MOIs (1, 3, 10)	\	↓cell viability	(30)
T98, U138	4 mM/72 h	TMZ/50 μM/72 h	MGMT	↓cell growth, ↓migration, ↑apoptosis, ↑autophagy, ↓xenograft growth	(31)
U87	4 mM/72 h	MSCs-TK	\	↓cell viability, ↑apoptosis, ↓xenograft growth, ↓survival	(32)
D384, T98	D384: 5 mM/48 h T98: 2.5 mM/48 h	D384: TMZ/0–20 μM/24 h T98: TMZ/0–500 μM/24 h	\	↓proliferation, ↓clonogenic capacity	(33)
D384, T98	D384: 5 mM/48 h T98: 2.5 mM/48 h	Radiation/4 Gy	\	↓proliferation, ↓clonogenic capacity	(33)
U87MG, Hs683, DBTRG-05MG	125 μM/72 h/144 h	TMZ/100 μM/72 h/144 h	Nrf2 signaling	↓cell-cycle progression, ↑apoptosis	(34)
U251, T98	0.5 mM/48 h	NY-ESO-1	NY-ESO-1	\	(35)
A172, U373, U138, U87, SW1783	0–5 mM/96 h	BCNU/0–125 μg/ml/96 h	H3, H4	↓proliferation, ↓cell-cycle progression, ↑apoptosis	(23)
C6	0.5 mM/48 h	Radiation/0–8 Gy	Bax/Bcl-2	↓cell viability	(36)

VPA, valproic acid; TMZ, temozolomide; ACNU, nimustine; MGMT, O6-methylguanine-DNA methyltransferase; EHV-1, equine herpesvirus type 1; BCNU, 1, 3-bis (2-chloroethyl)-1-nitrosourea.

↓ -Inhibition, ↑ -Promotion.

**Table 3:** Clinical implications of VPA in glioma patients.

Patients	Concentration (VPA)	Other Therapeutic Agents	Clinical Implications	Ref.
38 children with DIPGs or HGGs	15 mg/kg/day	Radiation: 50.4–54 Gy	↑EFS, ↑OS	(37)
112 patients with HGGs	800 mg/day	TMZ: 75 mg/m^2^/day Radiation: 60 Gy	↓hair loss, ↑OS, ↑PFS	(38)
165 GBM patients	10–15 mg/kg/day	Radiation: 18 Gy Carboplatin: 175 mg/m^2^ Vincristine: 1.5 mg/m^2^	↑median event-free survival, ↑median survival	(39)
44 glioma patients	10 mg/kg/day in week 1 20 mg/kg/day in week 2	\	↑median overall survival, ↓toxicity	(40)
2379 HGG patients	≥84 DDD (1,212) <84 DDD (1,167)	TMZ: 75 mg/m^2^/day	↑OS, ↓hazard ratio	(41)
359 glioma patients (WHO II-IV)	GBM patients (0.49–1825 g) Grade II/III patients (10.5–4,106.25 g).	TMZ: 75 mg/m^2^/day	↑OS (GBM) ↓histological progression, ↑PFS (Grade II/III)	(42)

VPA, valproic acid; DIPG, diffuse intrinsic pontine glioma; HGG, high-grade glioma; EFS, event-free survival; OS, overall survival; TMZ, temozolomide; PFS, progression-free survival; DDD, defined daily dose.

↓ -Inhibition, ↑ -Promotion.

## 2 Individual Anti-Tumor Effects of VPA in Glioma

### 2.1 Involvement With Cellular Activities

#### 2.1.1 VPA Inhibited Glioma Cell Proliferation, Migration, and Invasion

Cell proliferation, one of the important physiological functions of tumor cells, is the basis of growth, development, reproduction, and heredity (43). Moreover, cellular migration and invasion are also the most important features of malignant tumors, mainly involved in cancer metastasis (44). In most glioma cells and glioma stem cells (GSCs), VPA repressed cell viability in a dose- and time-dependent manner (2-10 mM for 48, 72, and 96 h) at varying degrees due to tumor heterogeneity (13, 17, 20). Subsequently, Trypan Blue dye exclusion assay was utilized to detect proliferative rates. Treatment with VPA induced a statistically significant reduction of the cell growth, ranging from 10% to 40%, with an increase in the LDH release (16, 17). Benítez et al. further validated the particular correlation between the decreased cell proliferation and increased cell death (21).

Indeed, VPA was verified to impair the migratory process of glioma cells according to Boyden chamber findings (16, 21). Meanwhile, the invasive capability of glioma cells was also influenced by VPA treatment (18, 45). As is well-known, the epithelial–mesenchymal transition (EMT) process and matrix metalloproteinases (MMPs) are vital for migration and invasion (46, 47). Concluding from results of Western blot and immunofluorescence, VPA treatment potentiated downregulation of Snail1 and Twist1 levels and relocalization of E-Cadherin, thus inactivating the EMT process (16). Ryu et al. also found that MMP-2 and MMP-9 were knocked down by VPA (18). Therefore, VPA exerted inhibitory effects on cellular proliferation, migration, and invasion in glioma *via* the EMT process and MMPs.

#### 2.1.2 VPA-Induced Glioma Cell-Cycle Arrest and Apoptosis

Cell cycle refers to the whole process that the genetic material of a cell is duplicated and equally distributed to the two daughter cells (48). It could be modulated by gene mutations and physical and chemical factors, which is indicative of apoptosis (49). Cell apoptosis is an active process of the orderly death of cells controlled by genes to maintain the stability of the internal environment for better adaptation to the living environment (50). To explore whether VPA had an inhibitory effect on cell cycle, DNA flow cytometric analyses and immunocytochemistry were carried out (20, 21). Obviously, VPA treatment could increase the population at the G0/G1 phase and decrease the population at the S phase, indicating that VPA induces G0/G1 arrest in malignant glioma cell lines (20). A growth arrest process is also involved in the decrease of cell proliferation, together with cell death and inhibition of cell migration (21). However, the cell-cycle-related proteins and mechanisms have not been detected, which appeals to additional experiments.

The apoptosis induced by VPA was measured by flow cytometry Annexin V-FITC/PI and TUNEL staining. Zhang et al. found that apoptotic rates of glioma cells were induced by VPA in a dose-dependent manner (17, 18). Further functional investigation illustrated that VPA upregulated expression of cleaved caspase 3 and cleaved caspase 9. Moreover, the expression of Bcl-2 family protein Bax and Bak was increased, whereas Bcl-2, Bcl-xl, and Mcl-1, the anti-apoptotic members, were decreased by VPA treatment (13, 17). Additionally, the release of cytochrome c from the mitochondria under VPA treatment has also been increased. The expression of apoptosis-inducing factor (AIF) and poly ADP-ribose polymerase (PARP) was also upregulated (17). Moreover, Chen et al. found that VPA treatment mildly suppressed the expression of JNK1 and increased the expression of phospho-JNK1 and phospho-ERK1/2, but had no effect on the expression of ERK1/2 (18). However, inhibition of JNK1 and/or ERK1/2 reversed the VPA-induced cytotoxicity and changes in apoptosis (18). Similarly, ERK and Akt proteins were phosphorylated, indicating that their activities were induced but GSK3β activity was inhibited, because of increased GSK3β phosphorylation (17). In summary, VPA promoted cell-cycle arrest at the G0/G1 phase, thus inducing apoptosis *via* activation of the mitochondria-mediated apoptosis and ERK/Akt pathway.

#### 2.1.3 VPA Was Involved in Cellular Autophagy and Angiogenesis

Autophagy is a highly conserved process that is essential for cell survival, host defense, and energy consumption (51). Autophagy in cancer is often described as a “double-edged sword”, which either promotes tumor survival under microenvironmental stress and increases growth and aggressiveness or suppresses tumorigenesis *via* its quality control function (52). Results of electron microscopy (EM), MDC staining, and GFP-LC3-labeling analyses revealed that autophagic vacuoles increased under treatment with VPA (13, 20). Since LC3-II is closely associated with the membrane of autophagosomes and p62 is a selective autophagy adaptor/receptor, binding ubiquitinated proteins and LC3 for engulfment, the expression of LC3-II and p62 was examined by Western blot analysis (53). Han et al. found that p62 expression was downregulated, while LC3-II expression was obviously upregulated in glioma cells treated with VPA (13). Similar promotion of autophagy could also be seen for Beclin-1 (20). Subsequently, p-Akt/Akt and p-mTOR/mTOR expression was apparently downregulated, thus inhibiting the Akt/mTOR pathway to promote autophagy (13). All these data suggested that VPA facilitated the induction of autophagy *via* the Akt/mTOR signaling pathway.

Angiogenesis refers to the formation of new blood vessels from existing capillaries or veins behind capillaries, which plays an important role in the development and metastasis of tumors (19). VPA reduced vascular endothelial growth factor (VEGF) secretion of glioma cells in a dose-dependent manner under both normoxic and hypoxic conditions. VPA was also found to inhibit tube formation in the angiogenesis assay. *In vivo*, treatment with VPA combined with irinotecan reduced the number of vessels expressing factor VIII in the brain tumor model. Therefore, VPA inhibited glioma angiogenesis by direct inhibition of endothelial cell proliferation and tube formation and indirectly decreased secretion of VEGF by glioma cells (14). Above all, VPA inhibited angiogenesis *in vitro* and *in vivo* targeting VEGF (**Figure 1** and **Table 1**).

### 2.2 Signaling Pathways and Molecular Targets

#### 2.2.1 Signaling Pathways

Several signaling pathways have been verified to participate in the initiation and progress of malignant tumors (54, 55). In addition, all these pathways have been involved in proliferation, migration, invasion, cell-cycle progression, apoptosis, autophagy, and angiogenesis, which could also be therapeutic targets for multiple therapies, especially for chemotherapy (56, 57).

In VPA treatment, VPA activated the Akt/mTOR signaling by decreased expression of p-Akt/Akt and p-mTOR/mTOR in U251 and SNB19 (13). While Zhang et al. reported that incubation with VPA increased phosphorylation of ERK and Akt in ERK/Akt signaling in U87, thus in turn inhibiting GKS3β activation by the induction of GKS3β phosphorylation (17). Further loss-of-function experiments illustrated that inhibitors of MAPK and PI3K pathways abolished apoptotic induction of VPA, but GSK3β inhibitor mimicked effects of VPA (17). Furthermore, p-JNK1 and p-ERK1/2 were also increased by VPA, while inhibition of JNK1 and/or ERK1/2 partially reversed the VPA effects, involved in MAPK signaling (18, 20). Moreover, DNA methylation changes of Wnt pathway-related genes and transcriptional activity of the β-catenin/TCF complex were obviously induced by VPA *via* a TOP/FOP flash reporter assay (16). Additionally, VPA treatment resulted in an enhancement of 5-HT2A receptor-stimulated PI hydrolysis (25). Hence, Akt/mTOR signaling, ERK/Akt signaling, MAPK signaling, Wnt/β-catenin signaling, and 5-HT2A signaling play vital roles in the functional activities of VPA.

#### 2.2.2 Molecular Targets

In addition to main signaling pathways, molecular targets also participated in functional activities of VPA. As one of the most common HDACIs, VPA caused significant time-dependent changes in histone deacetylase (HDAC) 1, 2, and 3 (22). The mRNA expression of the melatonin 1 (MT1) receptor and methyl CpG binding protein 2 (MeCP2) was also decreased by VPA (22, 24). At the same time, histone 3 (H3) and H4 acetylation were induced by VPA (21, 23). Rincón Castro et al. also detected the upregulation of brain-derived neurotrophic factor (BDNF) and glial cell line-derived neurotrophic factor (GDNF) in VPA activities (24). Apart from these, VPA reduced VEGF secretion of glioma cells in a dose-dependent manner under both normoxic and hypoxic conditions (14). Moreover, incubation with VPA markedly increased the expression level of CD44 and CD56 (26). All these findings manifested that VPA mainly exerted its biological activities *via* signalings and molecular targets mentioned above, providing evidence for pre-clinical experiments (**Figure 2** and **Table 1**).

## 3 Synergistic Effects of VPA With Therapeutic Agents in Glioma

### 3.1 VPA-Adjuvant Chemotherapy

#### 3.1.1 VPA and Temozolomide (TMZ)

TMZ, a 3-methyl derivative of mitozolomide, is the first-line chemotherapy drug of patients with gliomas, which easily pass through the blood–brain barrier (58). Despite its primary efficiency in glioma treatment, drug resistance is inevitable in patients with high O^6^-methylguanine-DNA methyltransferase (MGMT) (59). Gathering experiments have suggested that the combination of VPA with TMZ has combined or enhanced antitumor effects in glioma. For cytotoxic response, the combination of VPA and TMZ suppressed the survival rate and migration of glioma cells compared with that of the TMZ alone, which verified the sensitivity of VPA (28, 31, 33). More importantly, the combination induced apoptotic cell death, accompanied by enhanced DNA damage, intracellular GSH depletion, ROS production, and mitochondrial transmembrane potential disruption, *via* upregulation of Bax/Bcl-2 and cleaved caspase-3/caspase-3 (31, 34). Additionally, autophagic effects could also be activated by the combination of VPA and TMZ (31).

#### 3.1.2 VPA and ACNU/BCNU

ACNU and BCNU, referred to as common nitrosourea alkylating agents, have affinity to the blood–brain barrier. Researchers have verified their direct or indirect inhibitory effects on several tumors, including brain tumors, lung cancer, and gastric cancer (60, 61). Li et al. found that VPA enhanced the inhibitory effects of ACNU on the growth and apoptosis of MGMT-negative/positive cells, particularly in the MGMT-positive cells. Further mechanical investigation illustrated that VPA downregulated the expression of MGMT protein and promoted the methylation of MGMT promoter (28). As for BCNU, detailed analysis of combination of VPA and BCNU revealed that the synergistic effect was mainly reflected in cell growth inhibition and cell-cycle arrest rather than an increased programmed cell death (23).

#### 3.1.3 VPA and Luteolin

Luteolin is a natural flavonoid that could be extracted from traditional Chinese medical herbs (62). Indeed, it has been well established that luteolin has a variety of pharmacological effects, including anti-tumor, anti-inflammation, anti-oxidation, and immune regulation (63). Luteolin was verified to exert limited inhibitory effects on glioma cells, which contributes to application of VPA and luteolin. In this study, VPA enhanced the anticancer effects of luteolin in cell viability, colony formation, and migration. More importantly, VPA treatment induced cell-cycle arrest and cellular apoptosis *via* upregulation of p-PARP/PARP, cleaved caspase 3/caspase 3, and Bax/bcl-2. Moreover, VPA activated Akt signaling to promote autophagic response *via* accumulation of LC3-II and decrease of p62 (27).

#### 3.1.4 VPA and Gefitinib

Gefitinib, an oral tyrosine kinase inhibitor, selectively targeted the epidermal growth factor receptor (EGFR) (64). Due to its application in cancer treatment, its inhibitory effects, especially anti-angiogenesis, have been extensively acknowledged (65). However, a nontoxic concentration of VPA sensitized U87 and T98G glioma cells to gefitinib by inhibiting cell growth and long-term clonogenic survival *via* increased intracellular reactive oxygen species generation. In addition, the combination therapy induced autophagic response, accompanied by conversion of microtubule-associated protein-1 light chain 3-II (LC3-II), and degradation of p62 through activation of liver kinase-B1 (LKB1)/AMP-activated protein kinase (AMPK)/ULK1. Subsequent loss-of-function assay ascertained that knockdown of AMPK and ULK1 reversed the biological effect of the combination therapy-induced autophagy and growth inhibition (29). Therefore, VPA served as an adjuvant therapeutic agent for chemotherapy of glioma, contributing to subsequent exploration for novel chemotherapy drugs.

### 3.2 VPA-Adjuvant Radiotherapy

Radiotherapy, used in over 50% of cancer patients, mainly targets tumor tissues by using ionizing radiation with a little damage on normal tissues (66). However, emerging resistance to radiotherapy is the main obstacle in the clinical application (67). Therefore, it is an urgent need to combine an effective sensitizer with radiotherapy to obtain better outcome of tumor patients. Accumulating evidence showed that VPA enhanced radiation-induced cell death and the clonogenic formation at varying radiation doses from 0 to 6 Gy (33). Zhou et al. also explored that VPA induced apoptotic responses to irradiation by inhibiting Bcl-2 and increasing Bax at the mRNA and protein levels (36). However, the amount of trials focusing on VPA-adjuvant radiotherapy is too rare to verify its sensitization role in glioma.

### 3.3 VPA-Adjuvant Immunotherapy

Immunotherapy has made much progress with the cropping up of the immune checkpoint inhibitors (ICIs) (68). Apart from this, several immunotherapies, concerning dendritic cell, T lymphocytes, and oncolytic viruses, have been applied in the treatment of glioma (69). White et al. firstly evaluated the combination therapy of the lytic animal virus equine herpesvirus type 1 (EHV-1) with VPA. Surprisingly, VPA pretreatment promoted the infection and the yield of EHV-1, thus strengthening the ability spread laterally among cells (30). NY-ESO-1, an immunogenic cancer antigen, has been a specific target for immunotherapy. Sachie Oi et al. explored that VPA enhanced the induction of NY-ESO-1 by DNA-methyltransferase inhibitors (DNMTi). Further chromatin assays illustrated that the combination induced DNA demethylation, H3 Lys9 demethylation, and acetylation (35). Additionally, MSCs with high expression level of herpes simplex virus type I thymidine kinase (HSV-TK) were applied into glioma treatment. The results demonstrated that VPA and MSCs-TK synergistically induced cellular apoptosis of glioma cells *via* caspase activation, which was evaluated by TUNEL staining assay. Subsequent *in vivo* treatment also contributed to the same effects, including the suppression of tumor growth and survival time (32). Thus, the effects of VPA-adjuvant immunotherapy in glioma were so satisfactory that investigation should be carried out for more immunotherapy agents (**Figure 3** and **Table 2**).

## 4 Clinical Trials of Gliomas With VPA

A few clinical trials have illustrated that the clinical application of VPA showed ambiguous significance in glioma patients. For 44 glioma patients, only 3 patients developed somnolence, and average trough blood levels of VPA were below the safe standard, which verified that it had no severe toxicity (40). Further investigation performed by Francisco et al. revealed that compared to the non-treated group of six patients, median event-free survival and median survival of VPA group were much longer (15). Meanwhile, VPA seemed to serve as an adjuvant drug in glioma patients. Watanabe et al. explored that VPA contributed to survival improvement, including delayed hair loss and prolonged survival time (38). Similar improvements in prognosis of 165 GBM patients were also detected (39). In 38 children with DIPGs or HGGs, treatment of radiation and VPA could prolong event-free survival and overall survival, while only three patients developed thrombocytopenia, weight gain, and pancreatitis, respectively (37). Due to the different pathological grades and types of gliomas, more detailed research has been carried out. For GBM patients, VPA could reduce hazard ratio and improve overall survival (41, 42). For grade II/III glioma patients, prolonged PFS and decreased histological progression were correlated with positive VPA treatment (42). However, clinical significance of VPA treatment for recurrent diffuse intrinsic pontine gliomas (DIPGs) have not been testified (70). Therefore, though its safety has been initially confirmed, the clinical efficacy of VPA is still uncertain, appealing to a certain number of clinical studies (**Table 3**).

Additionally, application of VPA has been equipped with auxiliary benefits. Aiming at irreversible damage of radiotherapy, including apoptotic response of normal neuronal cells and neurocognitive deficits, VPA specifically protected hippocampal neurons from radiation-induced damage *in vivo* and *in vitro* (71). More concretely, VPA improved radiation-related hair loss in 112 glioma patients (39). Castro et al. also attributed the neuroprotective properties of VPA to modulation of BDNF, GDNF, and melatonin receptors (24), while there were still a few adverse effects, mainly in psychiatric, neurological, gastrointestinal, hematopoietic, and metabolic disorders (72). Firstly, hyperammonemia induced by VPA treatment would lead to unpredictable damage of nervous system, contributing to psychiatric disorders and neurological disorders, including cognitive dysfunction, Parkinsonism, emotional instability, insomnia, and neurasthenia (73–76). The defects of neural tube and axial skeleton further verified the role of VPA as a teratogen (77). Secondly, VPA would induce nausea, vomiting, indigestion, diarrhea, and constipation in a certain proportion of patients receiving VPA treatment (78). Thirdly, VPA might alter hematopoietic homeostasis for occurrence of thrombocytopenia and megakaryocyte dysplasia (38, 79). Fourthly, endocrine disturbances and subsequent weight gain were also main side effects of VPA (80). In addition, allergic symptoms, fever, hearing loss, menstrual disorders, and damage of liver and kidney function could also be observed (72, 81). Of course, stopping oral VPA and applying symptomatic drugs would be a great option for these related clinical symptoms (37).

## 5 Discussion

The reviewed data have provided supporting evidence for application of VPA as a therapeutic agent in glioma treatment. VPA was involved in several cellular activities, including cell proliferation, migration, invasion, cell-cycle arrest, apoptosis, autophagy, and angiogenesis. These biological actions focused on several signaling pathways, including Akt/mTOR, ERK/Akt, JNK1, ERK1/2, Wnt pathway, and 5-HT2A signalings. Apart from these targets, molecules like HDAC 1, 2, and 3, MeCP2, H3, H4, VEGF, CD44, and CD56 also played vital roles in treating glioma. Meanwhile, VPA was also verified to be an adjunctive agent in the treatment of chemotherapy, radiotherapy, and immunotherapy. Of course, a few clinical trials demonstrated that VPA improved survival of glioma patients. Surprisingly, knockdown of SEL1L, a crucial protein involved in homeostatic pathways, cancer aggressiveness, and stem cell state maintenance, increased VPA sensitivity to glioma (82). All these evidence have confirmed the therapeutic role of VPA in glioma treatment.

However, results of a few experiments were contrary to findings above. For example, Riva et al. reported that VPA induced the genome-wide DNA methylation profile and the differentiation behavior to elevate the sensitivity of VPA, while not TMZ (83). However, incubation of VPA promoted secretion of amphiregulin (AR), facilitating TMZ resistance (84). In addition to this, Han et al. found that VPA inhibited the Akt/mTOR pathway by reducing the expression of p-Akt/AKT and p-mTOR/mTOR in glioma cell lines U251 and SNB19 (13), while in the study conducted by Zhang et al., VPA could increase the phosphorylation of ERK and Akt in the ERK/Akt pathway in U87 (17). The reason for these differential expressions might be attributed to tumor heterogeneity, origin, gene drift, and experimental conditions. Therefore, more experimental and clinical investigation should be carried out for qualification of the inhibitory role of VPA.

Furthermore, many other signaling pathways and molecular targets have been detected in the functional processes of VPA in other tumors. For example, the STAT3/Bmi1 pathway could be modulated by VPA to increase the sensitivity of gemcitabine to pancreatic cancer cells (10). VPA also triggered the EMT process of colorectal cancer cells targeting Snail *via* the Akt/GSK-3β pathway (85). In addition, HIF-1α and Survivin played significant roles in the activities of VPA (86, 87). Gathering evidence demonstrated that synergistic antitumor effects of VPA and other therapeutic agents have been explored. The combination therapy of VPA and simvastatin sensitized prostate cancer cells *via* YAP inhibition (6). VPA and Arsenic Trioxide were verified to induce cell-cycle arrest at the G2/M phase and apoptotic cell death in lung cancer (88). Moreover, VPA could also enhance anti-PD-L1 tumor immunotherapy in blocking myeloid-derived suppressor cell function (89). To our surprise, VPA was also involved in gene therapy and antiblastic therapy (90, 91), which might be directions for subsequent research. Hence, it is an urgent demand to explore more reliable signaling pathways and adjuvant therapeutic agents in biological activities of VPA.

Currently, more and more anti-epilepsy drugs (AEDs), including VPA, phenobarbital, carbamazepine, clonazepam, levetiracetam, lamotrigine, topiramate, and oxcarbazepine, have been concerned in studying tumors of CNS. Among these AEDs, VPA and levetiracetam exhibits superiority in the clinical application of brain tumor-related epilepsy (BTE), due to their sensitization of TMZ through MGMT-dependent or MGMT-independent mechanisms (92, 93). In addition, levetiracetam and VPA would be beneficial to verbal memory and cognitive function *via* downregulating excitatory amino acid transporter-2 expression (94, 95). However, further evidence-based guidelines demonstrated that levetiracetam is the best first-line agent for BTE patients due to its efficacy and tolerability, especially in patients undergoing 5-ALA-mediated fluorescence-guided resection (FGR) (96). Different from levetiracetam, VPA contributed to prolonged survival of glioma patients, particularly in glioblastomas (97). Therefore, prospective evaluation of VPA and levetiracetam treatment for glioma patients is warranted to confirm these findings.

Despite these deficiencies and prospects of VPA treatment in glioma, accumulating evidence demonstrated that VPA exerted inhibitory effects on glioma targeting several signaling pathways or molecules individually or with chemotherapy, radiotherapy, and immunotherapy, contributing to further exploration.

## Author Contributions

WH designed and wrote the manuscript, and drafted the schematic figures and tables. WG contributed to the overall design, supervision of the work, and essential reading. All authors contributed to the article and approved the submitted version.

## Conflict of Interest

The authors declare that the research was conducted in the absence of any commercial or financial relationships that could be construed as a potential conflict of interest.

## Publisher’s Note

All claims expressed in this article are solely those of the authors and do not necessarily represent those of their affiliated organizations, or those of the publisher, the editors and the reviewers. Any product that may be evaluated in this article, or claim that may be made by its manufacturer, is not guaranteed or endorsed by the publisher.

## Abbreviations

VPA, valproic acid; OS, overall survival; PFS, progression-free survival; HDACIs, histone deacetylase inhibitors; GSCs, glioma stem cells; EMT, epithelial–mesenchymal transition; MMPs, matrix metalloproteinases; AIF, apoptosis-inducing factor; PARP, poly ADP-ribose polymerase; EM, electron microscopy; VEGF, vascular endothelial growth factor; HDAC, histone deacetylase; MT1, melatonin 1; MeCP2, methyl CpG binding protein 2; H3, histone 3; BDNF, brain-derived neurotrophic factor; GDNF, glial cell line-derived neurotrophic factor; TMZ, temozolomide; MGMT, O^6^-methylguanine-DNA methyltransferase; EGFR, epidermal growth factor receptor; LC3-II, light chain 3-II; LKB1, liver kinase-B1; AMPK, AMP-activated protein kinase; ICIs, immune checkpoint inhibitors; EHV-1, equine herpesvirus type 1; DNMTi, DNA-methyltransferase inhibitors; HSV-TK, herpes simplex virus type I thymidine kinase; DIPG, diffuse intrinsic pontine glioma; AED, anti-epilepsy drug; BTE, brain tumor-related epilepsy; FGR, fluorescence-guided resection; AR, amphiregulin.
